# Quantum Gas-Enabled Direct Mapping of Active Current Density in Percolating Networks of Nanowires

**DOI:** 10.1021/acs.nanolett.3c04190

**Published:** 2024-01-23

**Authors:** Julia Fekete, Poppy Joshi, Thomas J. Barrett, Timothy Martin James, Robert Shah, Amruta Gadge, Shobita Bhumbra, William Evans, Manoj Tripathi, Matthew Large, Alan B. Dalton, Fedja Oručević, Peter Krüger

**Affiliations:** † Department of Physics and Astronomy, School of Mathematical and Physical Sciences, University of Sussex, Brighton BN1 9QH, United Kingdom; ‡ Physikalisch-Technische Bundesanstalt, 10587 Berlin, Germany

**Keywords:** transparent conductive materials, silver nanowires, percolation networks, active current density imaging, quantum technology, ultrasensitive magnetometry

## Abstract

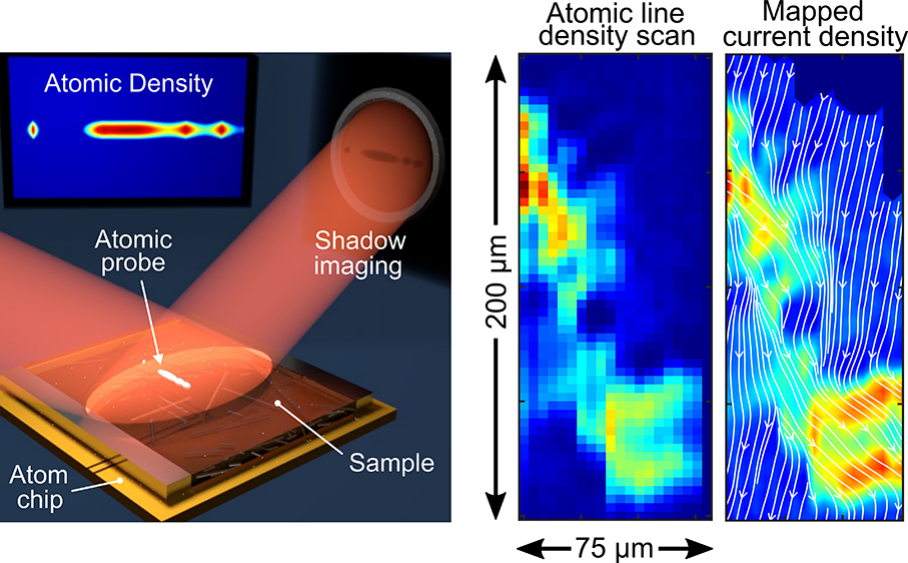

Electrically percolating nanowire networks are among the most promising candidates for next-generation transparent electrodes. Scientific interest in these materials stems from their intrinsic current distribution heterogeneity, leading to phenomena like percolating pathway rerouting and localized self-heating, which can cause irreversible damage. Without an experimental technique to resolve the current distribution and an underpinning nonlinear percolation model, one relies on empirical rules and safety factors to engineer materials. We introduce Bose–Einstein condensate microscopy to address the longstanding problem of imaging active current flow in 2D materials. We report on performance improvement of this technique whereby observation of dynamic redistribution of current pathways becomes feasible. We show how this, combined with existing thermal imaging methods, eliminates the need for assumptions between electrical and thermal properties. This will enable testing and modeling individual junction behavior and hot-spot formation. Investigating both reversible and irreversible mechanisms will contribute to improved performance and reliability of devices.

Over the last few decades percolating networks have attracted much interest, due largely to their increasingly important role in the development of transparent conductors^1^ and flexible thin-film transistors,^2^ as well as chemical^3^ and nanobio^4^ sensors. To characterize the relationship between the microscopic structure and the macroscopic physical properties of these networks, the percolation theory is commonly employed. Through a statistical approach, this theory has been successful in describing observed scaling laws, and counterintuitive responses for network properties such as electrical conductance and maximum voltage drop.^5−7^ These models play a crucial role in optimizing the network’s performance for various applications. The tunability of key experimental network parameters can, in turn, be used to test the theoretical models. In the study of electrical percolating networks, arguably the biggest remaining challenge is the absence of a noninvasive experimental technique capable of directly unveiling a spatially resolved current density pattern and its often nonlinear, dynamic changes when an electrical voltage is applied across the network. Such a tool would be advantageous where usual statistical models are inadequate, such as in identifying clusters leading to device failure. When used in combination with existing techniques, it would enable in-depth studies of the interplay among electrical, thermal, and surface properties, thereby bridging the gap between macroscopic network properties and microscopic observations. The absence of a viable direct microscopic current density imaging technique has stimulated alternative indirect measurements of the current flow. Instruments such as scanning electron microscopes, and atomic force microscopes (AFM) equipped with conductive, Kelvin-probe, or magnetic force modules, provide detailed information on the topography, conductivity, and important surface properties.^8^ Recently, thermal imaging has been demonstrated as an efficient tool to record thermal maps during active current flow in a network.^9−11^ This method revealed interesting mechanisms for hot-spot formation and clustering. For the interpretation of the measurements, the relationship between local current density and temperature relies on assumptions that only hold in the low-current-density limit. However, the thermal changes are detectable only when current densities are high, primarily due to the limited sensitivity of existing methods. In such high-current-density regimes, nonlinear thermoelectric interactions are significant, necessitating the use of more complex models.

Here we introduce a solution to the longstanding problem of current density mapping of random two-dimensional (2D) networks. Our approach exploits the capability of quantum gases, such as Bose–Einstein condensates (BEC), to detect ultralow magnetic fields. BEC microscopy (BEC-M)^12−14^ offers a unique combination of microscopic resolution, subnanoampere sensitivity, and tunable dynamic range. In addition, it is possible to map active current distributions in a single imaging shot rather than by time-consuming scans. Although these properties make this method an outstanding candidate for exploring the nonlinear phenomena in percolating networks, the required performance levels have not been reached so far.

In this paper, we analyze the feasibility of BEC-M for the study of electrical percolating networks. We report on benchmark experiments conducted on a microfabricated planar reference structure, demonstrating a substantial improvement over the previously reported performance. Then, at this performance level, we demonstrate the feasibility of the method for current density mapping in percolating networks. This is accomplished by simulating BEC-M data obtained from random nanowire networks with varying wire densities. We also discuss the key advantages over AFM. Furthermore, using a single junction model, we show how BEC-M has the potential to deepen our understanding of the thermoelectric interplay in these networks. This interplay underpins critical phenomena such as self-heating and the reconfiguration of current pathways.^10,11^

In the context of nonlinear percolating networks,^7,10^ we outline how the technique can be used to study nonlinear phenomena and observe dynamics including reversible as well as irreversible mechanisms (see example shown in Figure 4a).^11,15,16^ Finally, we experimentally demonstrate the suitability of BEC microscopy for nanostructured samples using a network of carbon nanotubes as an example.

Ever since their theoretical prediction in 1924^17^ and their first experimental observations in dilute alkali gases in 1995,^18^ BECs have garnered significant interest in quantum sensing applications, primarily because they outperform their classical counterparts in numerous aspects.^12,19−21^ BEC-M has been introduced^12,22−25^ as a technique to measure the local variation in the magnetic field. The method relies on atom chip technology,^26^ which enables the precise control and manipulation of BECs near surfaces. Small changes in the local magnetic field cause density variations of the atomic gas, which can be directly related back to variations in the current flow. This effect has been measured in lithographically fabricated (evaporated)^25^ and electroplated wires^27^ as well as in microstructured patterns.^13^ It has also been used to observe the nematic transition in iron pnictide, a high-temperature superconductor.^28^

The working principle of BEC-M involves neutral atoms in an ultrahigh-vacuum (UHV) environment. The atoms are initially laser cooled, then magnetically trapped and further cooled by forced evaporation below the critical temperature for Bose–Einstein condensation (typically hundreds of nanokelvins).^18^ In our experiment, the magnetic trapping relies on precisely controlled currents in various components such as atom chips, printed circuit boards (PCB), millimeter-thick wires, and coils to generate a magnetic field with a desired spatial distribution. This flexibility enables versatile adjustment of the shape and position of the atomic probe. We typically prepare a highly elongated ultracold atomic cloud on an atom chip for microscopy measurements, as depicted schematically in Figure 1a.

**Figure 1:**
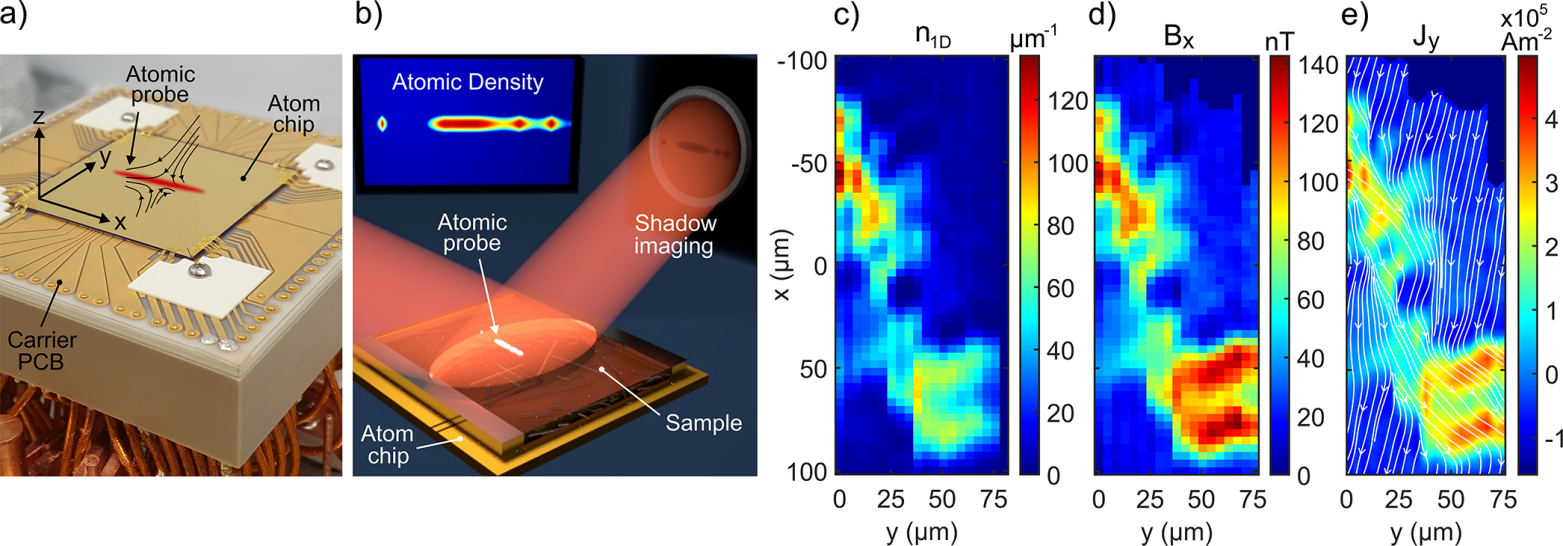
(a) Trapping structure (PCB and atom chip) and illustration of magnetic field lines in the *y*–*z* plane of a magnetic quadrupole forming a trap used for the radial confinement of the atomic probe. (b) BEC-M operation scheme. (c) A raster scan of atomic line densities *n*_1D_(*x*) measured over a planar microfabricated conductor. (d) Magnetic field distribution and (e) current density calculated from (d). The streamlines represent current flow with their density being proportional to the current density *J*_*x*_. To aid the visualization of the inhomogeneous flow, the current density *J*_*y*_ has been multiplied by a factor of 1000.

For microscopy, a sample of interest is placed between the trapped atoms and the trapping structure. Due to the intrinsically small energy scales in BECs, even magnetic fields on the picotesla scale emerging from the sample can alter the magnetic trapping potential sufficiently to impact the confinement of the atoms. In response, the atomic wave function is distorted, and the modified atomic density distribution can be captured using conventional imaging techniques.

Figure 1b shows a schematic illustration of the BEC-M operation. The field distribution emanating from the sample can be reconstructed along the quasi-one-dimensional (1D) probe (*x* direction) from a single-shot image taken in a fraction of a millisecond. A 2D-field map can be created by raster scanning the probe position along the orthogonal direction (*y*) across the sample surface. In our demonstration implementation, each field recording involves the recreation of the atomic probe, due to the destructive nature of the imaging process, with a duty cycle on the order of seconds.

Here, we demonstrate experimentally an improvement in the state of the art sensitivity of BEC-M, to the point that the method becomes feasible for current density mapping in nanowire networks. For this demonstration, our BECs were scanned over the same microfabricated planar conductor that served as a trapping wire for the atoms, and we show how a current density map is inferred from measured atomic densities (see the Supporting Information for further details). The current inhomogeneity observed in this conductor arises from imperfections in the fabrication process. This measurement provides a benchmark for future measurements of other samples, including nanowire networks.

The atomic densities measured at each position were converted into line densities *n*_1D_(*x*), resulting in the 2D map shown in Figure 1c. From the line densities, the change in magnetic field component *B*_*x*_ can be approximated^29^ by 2*hν*_⊥_*a*_sc_/μ_B_·δ*n*_1D_(*x*), as shown in Figure 1d. Here *h* is Planck’s constant, ν_⊥_ is the radial trapping frequency, *a*_sc_ is the s-wave scattering length, and μ_B_ is the Bohr magneton. The current density *J*_*y*_ (giving rise to the *B*_*x*_ component of the magnetic field) is then calculated from the measured magnetic field distribution using an inverse method^30^ and is shown in Figure 1e.

The single shot noise floor for these measurements was 0.18 atoms/μm, which converts to a minimum detectable field in Figure 1d of δ*B*_*x*_ = 180 pT. The maximum field value of 140 nT indicates a dynamic range factor of 770. The sensitivity to current is 1.3 nA, assuming an infinitely thin wire as the source of the field. This is a valid approximation in the case of a random nanowire network as the distance between atoms and the wires is significantly larger than the wire thickness.

Interestingly, we have observed field gradients as large as 8 nT μm^–1^ (when averaged over 3 repeats). Such a gradient would allow for probing steps below 50 nm while detecting finite changes in the magnetic field (considering only the field detection limit). However, the accuracy of lateral positioning is currently limited by the noise level of the power supplies used for trapping.

The cloud size (radii of 290 nm radially and 34 μm longitudinally) determines the ultimate spatial resolution and the field of view. The resolution of BEC-M is limited by two parameters. Along *x*, the resolution is limited by optical diffraction, typically a few micrometers. Along *y*, the precise control of the atomic probe position and the radial confinement determines the effective spatial resolution. The orientation of the atomic probe and the scanning direction can be rotated by 90° if high resolution is required in both directions. By tightening the radial confinement (proportional to ), one can further improve the resolution, however, at the cost of reduced field sensitivity (proportional to ν_⊥_).

To put these performance values into context, it is worth comparing the sensitivities of all existing magnetometers. State-of-the-art magnetic field sensors are dominated already by quantum sensors, but there is a tradeoff between sensitivity and spatial resolution.^13,31^ The sensors with highest sensitivity operate in the millimeter to centimeter range, and sensors that can resolve field variation at the nanoscale have orders of magnitude lower sensitivity. For measuring DC magnetic fields emanating from direct current sources, our improved BEC-M offers the best performance in terms of sensitivity at the microscopic scale.

We now demonstrate the feasibility of the improved BEC-M through simulations on percolating networks of different wire densities under operating conditions similar to the measurements described in the previous section. We chose the parameters from silver nanowires, as they are a key contender for practical applications of the networks. Figure 2 shows random numerically generated wire networks (top panels) and the corresponding reconstructed current density distributions (bottom panels) for both a network near the percolation threshold and a high-density network. The network density is related to the transparency of the sample, which is a key quantity for transparent electrodes and touch screens.^32^ In the two cases presented here, the optical transmittance values are 90% and 80%, respectively.

**Figure 2:**
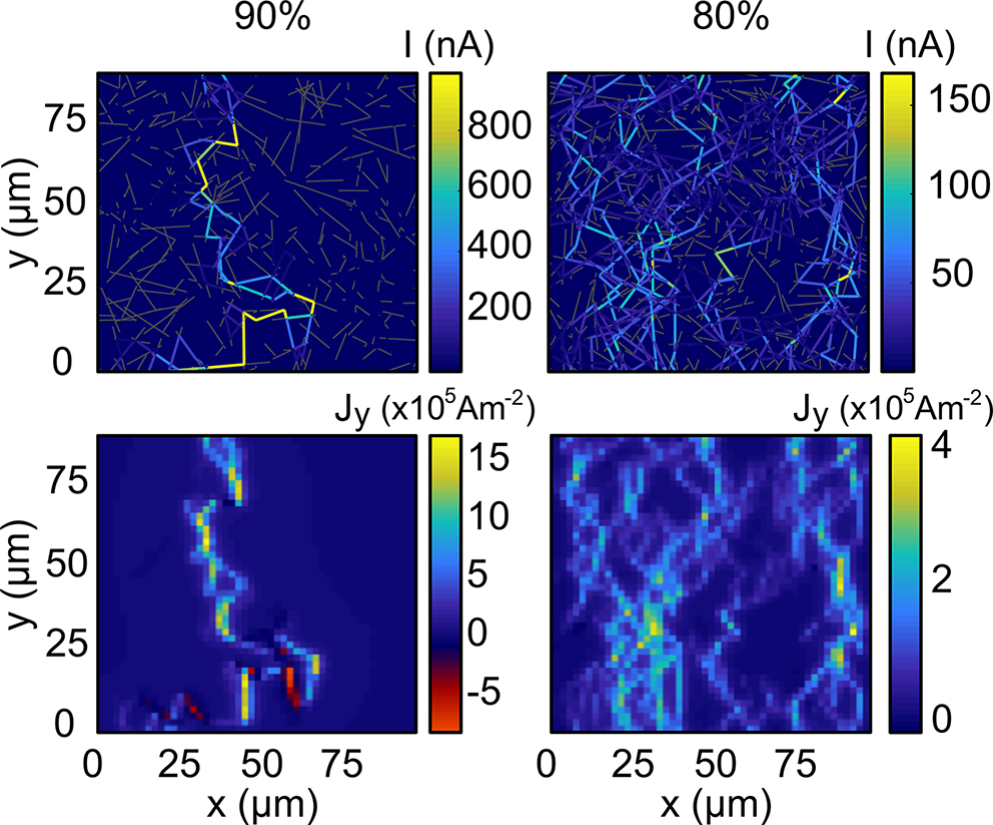
Simulation of randomly generated networks of silver nanowires, with 120 nm wire diameters, subject to a 1 μA input network current. The optical transmittances are 90% (left) and 80% (right). Colors indicate the current in each wire (no current in the dark gray wires). Lower panels show the reconstructed current density distributions from the respective networks using BEC-M, for a radial trapping frequency of ν_⊥_ = 1 kHz at an atom–surface distance of 1 μm. Current flows from the bottom to the top edge.

These examples show that it is possible to distinguish individual current paths and junctions even well above the percolation threshold for standard operating conditions of the BEC-M. One can also observe sections with the reverse direction of the current flow, information that other methods are not able to reveal. In the 80% optical transmittance case, hot spots are visible and the current paths can still be resolved.

To highlight the advantage of BEC-M (i.e., measuring active current flow as opposed to conductivity), we now compare a simulated BEC-M current density map to experimental data measured on a small section of a real nanowire network by atomic force microscopy. The topography scan (Figure 3a) and corresponding conductive (C-AFM) measurement (Figure 3b) indicate that these nanowires have approximately 30 nm diameter and typically exceed a length of 10 μm. Importantly, one can identify dead ends that do not carry any current in a live network. These dead ends appear as conductors, not distinct from the active current-carrying structures, in the C-AFM due to the circuit being closed by the path from the bias electrode to the AFM tip. It can be seen that the measured current value is very sensitive to the tip position relative to the nanowire axes, resulting in a discontinuous map. Therefore, the measured current cannot be expected to be proportional to the current in a live network. Besides this issue, the sensitivity and dynamic range are not appropriate to inform of the presence of hot spots in a network, especially of those forming at low bias voltages.

**Figure 3:**
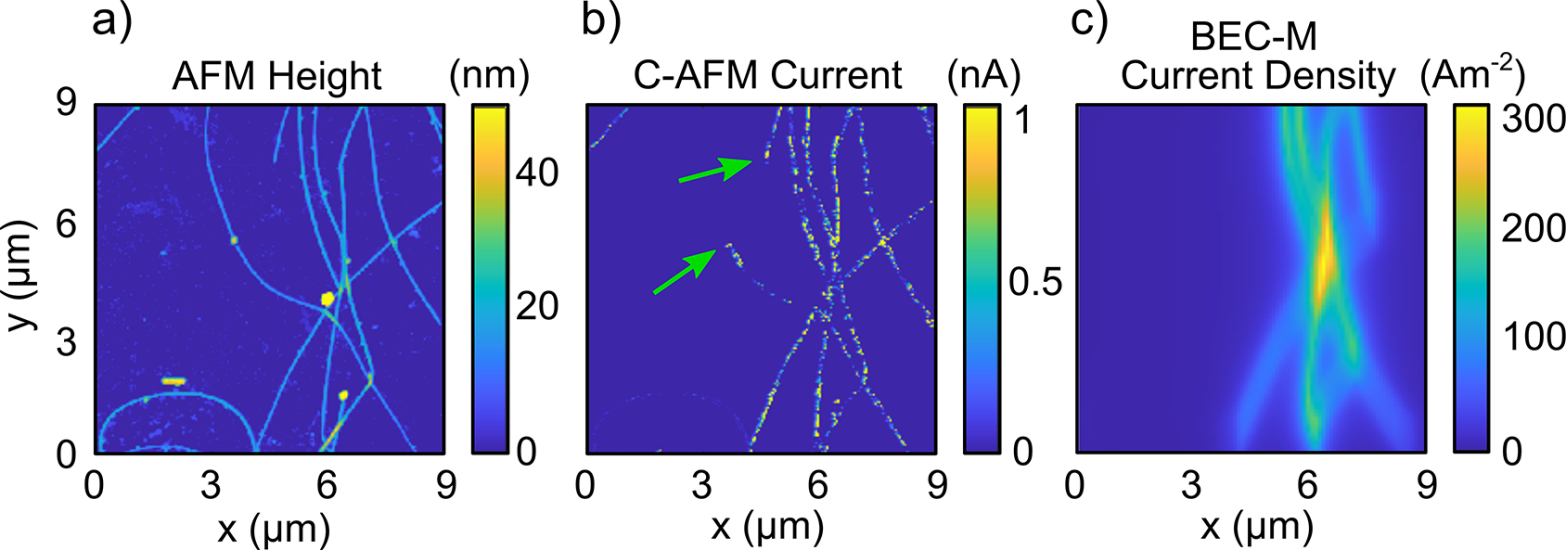
(a) Height map of a silver nanowire network section measured by AFM and (b) current map recorded by C-AFM, with the arrows indicating examples of dead ends. The discontinuous map of measured current appears as a sequence of dots. (c) Reconstructed active current density map from the simulation of BECs 100 nm above the network for the same topography for 100 nA input current. Trapping atoms at submicrometer distances from a surface poses a challenge due to the attractive Casimir–Polder force,^33^ but various approaches have been introduced to reduce the atom–surface distance near or below 100 nm.^34−36^

In contrast to the C-AFM measurement, the current density image obtained from the simulated BEC-M data for the same topography reveals the active current paths only and shows no false connection at dead ends. Absolute measurements of the current density can be evaluated if the topography is known and has a dynamic range at least 2 orders of magnitude above the minimum detection limit. Figure 3c was simulated by using the network topography in Figure 3a with bulk silver resistivity, assuming a typical junction resistance value of 100 Ω and 100 nA input current applied to the network section (via two electrodes at the top and bottom edges). This represents a realistic example of a section where a hot spot can form within a larger network. The presence of multiple junctions with resistances higher than a nanowire segment of equivalent area gives rise to the formation of hot spots, as is evident in the simulated current map.

The current is assigned to the network following Kirchhoff’s law and produces a magnetic field distribution at the location of the probe, which affects the atomic distribution. The current density is then recovered from the field by an inverse method.^30^

We now turn to studying percolating networks in terms of the thermoelectric interplay within and between their components. As an example of nontrivial thermoelectric behavior, see the heating measurement in Figure 4a, which demonstrates the nonlinear temperature-dependent resistance in a network and ultimately network failure. Even though microscopic observations of single-nanowire and single-junction resistances,^37^ as well as of networks by thermal imaging,^10^ have become available, no models succeed in explaining the non-Ohmic—also termed as super-Joule—behavior and its connection to the dynamic current path redistribution over networks. Furthermore, the rich dynamics at microscopic scales are not necessarily reflected in macroscopic observations, as in examples where substantial reconfiguration of the current does not lead to an appreciable change in the total resistance of the network.

**Figure 4:**
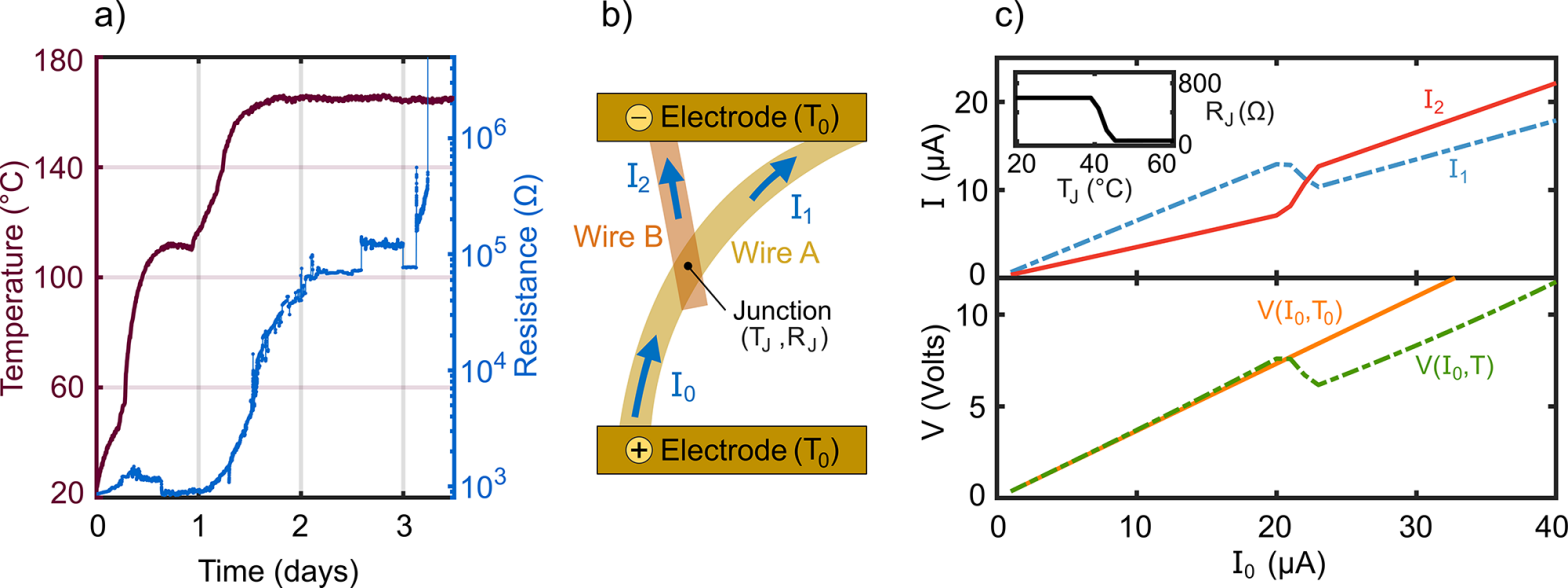
(a) Measurement of a nanowire network resistance while the sample was heated, exhibiting a nonlinear response. The abrupt increase in resistance in the final stage is irreversible and corresponds to network failure. (b) Schematic of the model setup, showing two wires crossing at a junction between two electrodes. The junction has a resistance *R*_J_ and a local temperature *T*_J_. (c) Currents *I*_1_ and *I*_2_ (upper plot) in the two upper wire sections of the circuit. The inset depicts the temperature-dependent junction resistance prescribed in the model. Voltage across the circuit (lower plot), showing the *I*–*V* characteristic of the circuit when taking into account the temperature dependence in the model *V*(*I*_0_,*T*) and when ignoring it, *V*(*I*_0_,*T*_0_). The abrupt change in voltage corresponds to a current path reconfiguration.

Here we introduce a model to show how such reconfiguration of the networks can be understood via Joule heating of the wires and a nonlinear junction response to the temperature. The numerical model consists of two wires in contact at a junction (Figure 4b). We assume the junction to have a temperature-dependent resistance, as shown in Figure 4c inset, exhibiting an abrupt drop in resistance *R*_J_(*T*_J_) at a certain temperature, consistent with the measurements of Bellew et al.^37^ Such a sudden drop in resistance could be a result of enhanced electrical contact due to material softening during heating, for example.

For a fixed current *I*_0_ between the electrodes we calculate the temperature and resistance along the sections of wire and the resulting currents using the 1D heat diffusion equation (see the Supporting Information for details). For certain parameters, we observe current path reconfiguration (Figure 4c upper panel) due to the nonlinear junction response, which brings the system into the regime of super-Joule heating.

The voltage across the circuit (*V*(*I*_0_,*T*) in Figure 4c lower panel) shows a behavior similar to that observed in Bellew et al.^37^ The linear *I*–*V* regime at low currents is followed by a significant drop, after which linearity is regained at larger currents. Such behavior is considerably different from the case where temperature dependence is not taken into account (*V*(*I*_0_,*T*_0_) in Figure 4c lower panel).

In contrast to thermal maps, which are proportional to the Joule heat, active current maps obtained by BEC-M at various current levels (as low as nanoamperes) enable quantitative analysis of the reconfiguration. The complete information to model percolating networks and work out individual junction behavior will be available from the combination of the two methods.

In the following, we describe our BEC-M system that provides the capability to perform microscopy on a series of samples, which is beneficial when aiming for a systematic comparison. We also demonstrate the application of the system to obtain experimental data from the nanostructured samples.

The atomic probe in BEC-M requires both a UHV environment and optical access during various stages of the experiment (such as laser cooling and imaging). We therefore implemented a scheme where initial trapping and probing take place in physically distinct regions, connected by atomic transport, on a single PCB (Figure 5a). This provides versatility on the optical properties of the samples and design flexibility for the imaging system and permits the use of a series of samples.

**Figure 5:**
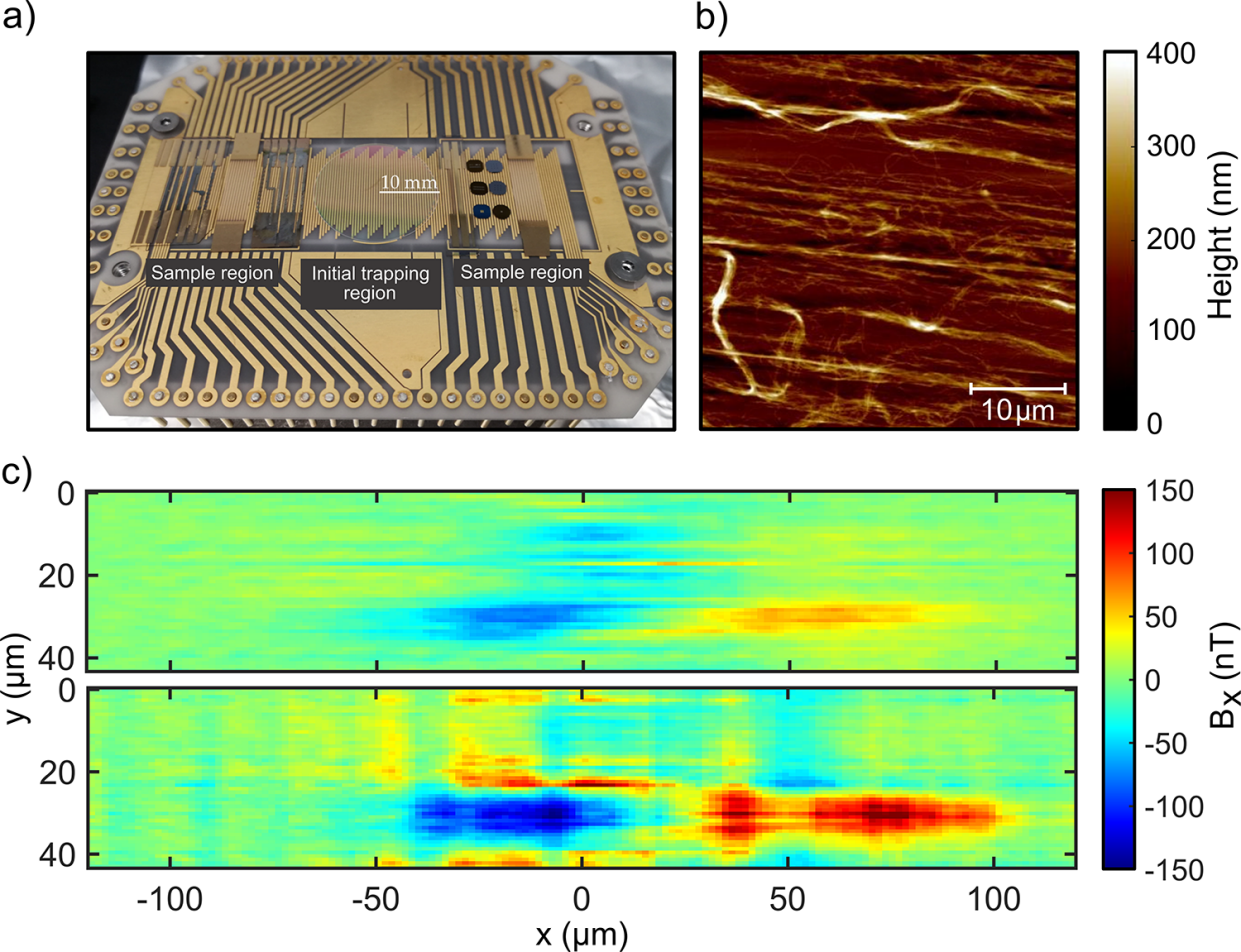
(a) Photograph of a PCB capable of magnetically transporting atoms from the initial trapping and cooling region to the samples, where BEC-M scanning takes place. (b) AFM height map of a representative (not the same as that scanned in (c) area of a carbon nanotube sample. The typical features that explain the observations of the magnetic field map in (c) are visible as individual nanotube fibers directed at various angles away from the dominant orientation of the fibers prescribing the main current flow. (c) Maps of the magnetic field arising from total currents of 50 mA (top) and 100 mA (bottom) being distributed across the carbon nanotube sample, measured by BEC-M. The trap shape (cloud radii of 41 and 1.3 μm in the longitudinal and radial directions, respectively), and the atom–surface distance (7.8 μm) were kept constant throughout the measurement.

Certain types of samples can be placed directly in the central atom trapping region, making the system and its optimization procedure simpler. To demonstrate the feasibility of BEC microscopy over nanostructured samples, we have fabricated a percolating network of carbon nanotubes (CNTs) on a substrate, as shown in Figure 5a. Even though this sample is neither transparent nor optically flat, we empirically found it possible to create an efficient magneto-optical trap suitable for BEC production, illustrating the general robustness of the method.

We have scanned the probe with and without current running through the CNT sample, and the difference is used to calculate the magnetic field generated by the sample as shown in Figure 5c. For higher current (50 mA in the upper panel, 100 mA in the lower panel), the redistribution of atoms in the trap, and therefore the magnetic field detected, is more pronounced. The structure seen in the magnetic field component parallel to the atomic probe arises solely due to current components in the orthogonal direction in the sample. Although the CNT sample is aligned approximately parallel with the probe (along the *x* direction), the off-axis current flow (i.e., deviations into the *y* direction) accounts for the BEC-M observations. Such structural features are typical in the CNT sample, as can be seen in Figure 5b, which shows an AFM topographic map of a representative region of the CNT sample.

In conclusion, we have experimentally demonstrated, and further analyzed through numerical studies, that the BEC-M technique is able to map current patterns at very low current levels—in the nanoampere range—where the sensitivity of thermal imaging methods is not sufficient. Our current density characterization will for the first time avoid assumptions regarding the link between thermal and conductive properties. Note here the importance of independently assessing current and temperature changes when their relationship is nonlinear. Combined with existing thermal imaging methods^9−11^ this relationship can be studied extensively, testing individual junction behavior and hot-spot formation in networks.

At high currents where network failure is likely, the microsecond scale imaging duration of BEC-M allows for stroboscopic observation of the network dynamics. Insights into irreversible evolution in the networks will enable a better understanding of material and device failure and, therefore, aid the development of devices with enhanced macroscopic performance. Beyond current density mapping the BEC-M also allows for studies of atom–surface interactions^33^ which may be of great interest for sensing applications with nanostructures.

## Data Availability

Data supporting this study are openly available from Figshare of the University of Sussex at DOI 10.25377/sussex.25029683.

## Supporting Information Available

Further details on the methodology, analysis, and specific parameters for the experimental demonstration BEC-M measurement on the microfabricated planar conductor, the nonlinear thermoelectric numerical model for investigating current path reconfiguration, and the experimental BEC-M scans over the carbon nanotube sample (PDF)
