# Supplementary material for: Quantum Gas-Enabled Direct Mapping of Active Current Density in Percolating Networks of Nanowires
Source: Nano Lett. 2024 Jan 23;24(4):1309–15. doi: 10.1021/acs.nanolett.3c04190 (PMC10835730; doi:10.1021/acs.nanolett.3c04190)
Supplement: Supplementary file 1 — nl3c04190_si_001.pdf [file nl3c04190_si_001.pdf]

Supporting Information for:

Quantum gas-enabled direct mapping of active current density in percolating networks of nanowires

Julia Fekete[1], Poppy Joshi[1], Thomas J. Barrett[1], Timothy Martin James[1], Robert Shah[1], Amruta Gadge[1], Shobita Bhumbra[1], William Evans[1,2], Manoj Tripathi[1], Matthew Large[1], Alan B. Dalton[1], Fedja Oručević[1], and Peter Krüger[1,2]

[1]Department of Physics and Astronomy, School of Mathematical and Physical Sciences, University of Sussex, Brighton, BN1 9QH, United Kingdom
[2]Physikalisch-Technische Bundesanstalt, 10587 Berlin, Germany

1 BEC-M Measurement Procedure

Here we describe in detail the BEC-M measurement procedure that was used for the experimental data presented in Figure 1 of the main text. First, an ultracold gas of rubidium-87 atoms in an ultra-high vaccuum (10^{-10} mbar) environment was prepared at a temperature of 1uK using standard techniques of laser cooling, magnetic trapping, and forced evaporative cooling [1]. The atoms were then trapped by the atom chip [2], which is comprised of a variety of planar conductors microfabricated using lithography and gold evaporation. Precisely controlled currents in the atom chip conductors produce a magnetic field of desired spatial distribution. Since atoms in correctly prepared (low-field seeking) magnetic quantum states are trapped in the regions of the lowest magnetic potential (proportional to the field magnitude), this allows for flexible manipulation of the atomic probe to vary its shape and position in three-dimensional space. Typically, currents of 300 mA to 1 A are passed through the atom chip conductors. After trapping, a further cooling phase brings the cloud below the transition temperature of 450 nK, producing a Bose-Einstein condensate (BEC) of approximately 100,000 atoms at a temperature of 200 nK. The resulting gas of atoms forms the atomic probe for subsequent microscopy measurements.

For the measurement presented in Figure 1 of the main text, the BEC is trapped by a conductor that is 200 μm wide and 2 μm thick, at an atom-surface distance of 1.4 μm. To perform microscopy, the probe is then scanned across the surface of the conductor in the y-direction over a total distance of 80 μm in 4 μm steps, as shown in Figure 1 below. This translational movement of the probe is achieved using an additional vertical external bias magnetic field. At each position, the 2D atomic column density distribution is recorded using standard absorption imaging techniques [3]. A brief (1 ms) period of free expansion is implemented between extinguishing all magnetic fields and capturing each absorption image, which simplifies the quantitative analysis of the images. When scanning the atomic probe, meticulous attention was given to maintaining the atom-surface distance (1.4 μm), the trap shape (22 Hz and 1.4 kHz in the longitudinal and radial directions, respectively), and field minimum (130 μT) throughout.

The measured 2D atomic column density distributions $n_{2\mathrm{D}}(x, z)$ are then converted into a series of 1D line densities $n_{1\mathrm{D}}(x)$, by integrating the signal along the z direction. Each line density profile then corresponds to a different y position, ultimately resulting in a 2D map in the $x - y$ plane. The deviations in atomic density in this map can be converted into a change in magnetic field component $B_x(x, y)$ using the approximation $2h\nu_\perp a_{sc}/\mu_\mathrm{B} \cdot \delta n_{1\mathrm{D}}(x)$, which is valid in the low density limit ($n_{1\mathrm{D}} < 100\,\mathrm{atoms}/\mu\mathrm{m}$) [4].

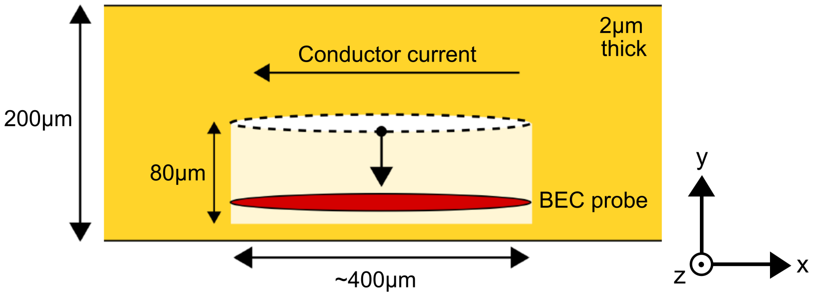

Figure 1: Schematic of the gold track used in the BEC-M demonstration. The atomic probe (red) was scanned over the surface in the light yellow region.

Here h is Planck's constant, $\nu_\perp$ is the radial trapping frequency, a_{sc} is the s-wave scattering length, and μ_B is the Bohr magneton. This magnetic field map is shown in Figure 1d of the main text.

The current density J_y that gives rise to the B_x component of the magnetic field can then be recovered from the measured field by using an inverse method based on the Green's function for the Biot-Savart law [5]. This method gives a unique solution with the assumption that the source current is restricted to 2D (i.e. does not have any variation along the z direction), which is a resonable assumption in this case. This results in the current density map shown in Figure 1e of the main text.

2 Nonlinear Thermo-Electric Model

Here we provide further details of the model used in Figure 4 of the main text, which investigates the thermo-electric interplay in a nanowire network. The model demonstrates a simple example of how current path reconfiguration in a network can be understood via Joule heating of the wires and a nonlinear junction response to the temperature.

The system considered is shown schematically in Figure 2a below, and consists of two wires (Wire A and Wire B) which overlap at a connecting junction, J. The variable input current I_0 is prescribed to flow from the positive electrode (E1) to the negative electrode (E2), and the relative currents I_0, I_1 and I_2 through each section will be distributed through the network according to the relevant resistances. The equivalent electrical resistance circuit for the model is shown schematically in Figure 2b. The lower and upper sections of Wire A have resistances R_0 and R_1, respectively, while Wire B has resistance R_2.

To include thermal effects in the model, we begin by prescribing a temperature-dependent resistance $R_\mathrm{J}(T_\mathrm{J})$ for the junction itself. We choose an abrupt drop in resistance at the junction from $600\,\Omega$ to $10\,\Omega$ as the temperature rises from $40\,°\mathrm{C}$ to $44\,°\mathrm{C}$. In the absence of extensive experimental characterization of $R_\mathrm{J}(T_\mathrm{J})$, we base our model on this assumption, as it is consistent with the measurements of Bellew et al.[6] This resistance characteristic is displayed in Figure 2c (solid line). Such a sudden drop in resistance could be a result of enhanced electrical contact due to material softening during heating, for example.

In addition to the junction, we also specify the resistances $R_i(T)$ of the remaining wire sections according to their geometry. For the model discussed here, we choose the material of the wires to be silver, and use the bulk resistivity value of $1.59\,\mu\Omega\,\mathrm{cm}$. The silver nanowires are of circular cross-section 60 nm in diameter, with lengths of 200 μm, 100 μm, and 80 μm corresponding the sections R_0, R_1, and R_2, respectively. The resistivity temperature coefficient of bulk silver of $0.0038\,\mathrm{K}^{-1}$ was used for all wire sections, and the resulting temperature dependence of the resistances $R_1(T)$ and $R_2(T)$ is shown in Figure 2c (dashed lines).

To solve the model, we allow a fixed current I_0 between the electrodes E1 and E2. After numerically discretizing all wires into small segments, we calculate the temperatures and resistances in each segment along the wires from the steady-state solution of the 1D heat diffusion equation. As the heat source, we

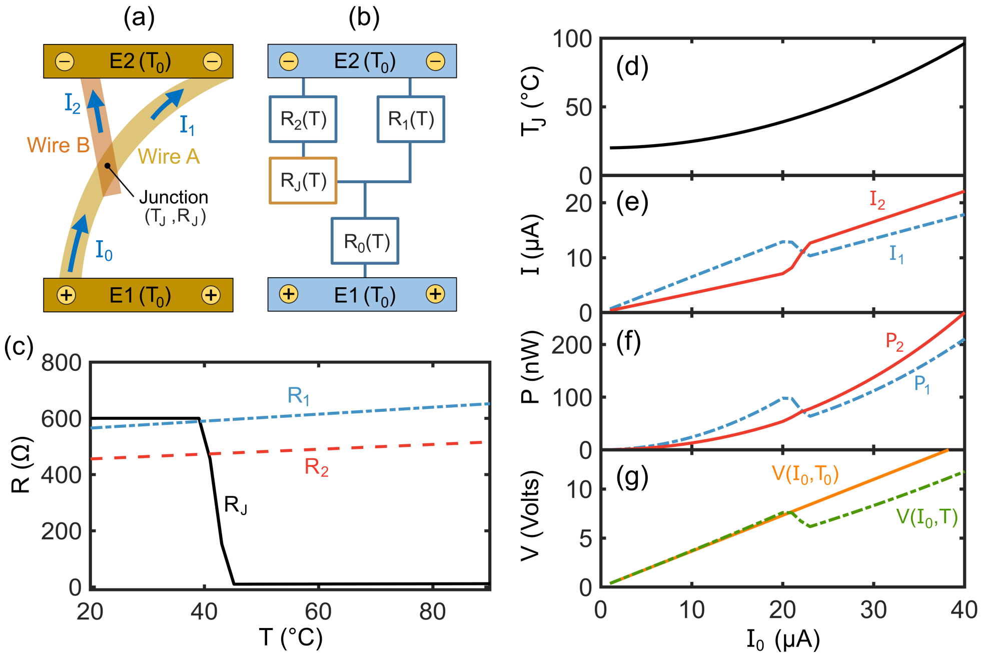

Figure 2: (a) Schematic of the model setup, showing two wires crossing at a junction between two electrodes. (b) Equivalent electrical circuit for the model, with each wire section having a temperature-dependent resistance. (c) Temperature-dependence of the wire sections and junction resistances. (d) Increasing junction temperature as a function of input current I_0. (e) Currents I_1 and I_2 and (f) power dissipation P_1 and P_2 in the two upper wire sections of the circuit. (g) Voltage across the circuit, showing the I-V characteristic of the circuit when taking into account the temperature dependence in the model $V(I_0, T)$, and when ignoring it $V(I_0, T_0)$.

use Joule heating due to the variable power dissipation in the conductors, and for the thermal boundary conditions we fix the temperature of the extreme wire ends at T_0=20 °C, so that the electrodes act as thermal baths. In this case, the steady state situation admits an analytic solution, for which the resulting temperature distribution along a wire is parabolic $T(x) = \gamma/2 \cdot x^2 - \gamma L/2 \cdot x + T_0$. Here, $\gamma = I^2R/\kappa V$, where κ is the thermal conductivity (419 W/m·K for silver), and V is the volume of the conductor. As the temperature rises due to power dissipation, the position-dependent resistances are then self-consistently adjusted accordingly. The junction then takes up the temperature of the local wire segment at all times.

The results of the model are shown in Figure 2d–g above. As the input current I_0 is increased, the temperature gradually rises due to power dissipation (Figure 2d). The ratio of currents in the two wire sections between E2 and the junction depends on I_0. For low values of I_0, Wire B carries less current than the upper section of Wire A ($I_2 < I_1$, shown in Figure 2e), due to the large junction resistance. However, for larger currents ($I_0 \sim 20\,\mu$A) the temperature rises to the threshold value of ∼40 °C, at which point the drop in junction resistance is induced. The nonlinear junction response reconfigures the current path, and $I_2 > I_1$.

This reconfiguration brings the system into the regime of super-Joule heating. Indeed, in contrast to the Joule heating power ($P_1 = R_1 I_1^2$) that scales with I_1^2, the power $P_2 = (R_2 + R_J)I_2^2$ exhibits a qualitatively different behaviour compared to I_2^2 (as shown in Figure 2f). Experimentally, thermal imaging provides maps of the Joule heating power, but it does not give access to resistance or current information in the network directly. By obtaining the active current maps by BEC-M, the complete information necessary to model the percolating network and to work out individual junction behaviour would be available from the combination of the two methods. The reconfiguration of the current paths, however, can be quantitatively measured

at various current levels over the entire network using the BEC-M alone, even at current levels as low as nanoamperes.

The voltage across the circuit [$V(I_0, T)$ in Figure 2g] shows a similar behaviour to that observed in Bellew et al. [6]. The linear I-V (Ohmic) regime at low currents and a slight deviation from it at medium current levels is followed by a significant drop after which linearity is regained at larger currents. Such behaviour is considerably different from the case where temperature dependence is not taken into account [$V(I_0, T_0)$ in Figure 2g].

3 CNT Measurement Details

Here we give specific experimental details regarding the BEC-M measurement over a carbon nanotube (CNT) sample, which was presented in Figure 5 of the main text.

The CNT sample was fabricated on a sample strip, with dimensions 18 mm long and 3.5 mm wide. The sample strip was situated on a thin (110 µm) substrate, that additionally serves as quarter waveplate for controlling laser polarisation during the initial atom trapping.

For these data, the atomic BEC probe consisted of ∼40,000 atoms at a temperature of 400 nK. The probe was oriented with the elongated axis along the x-direction, and then scanned across the current-carrying CNT sample surface over a range of 40 µm in steps of 780 nm along the y-direction. Throughout the scan, the trap shape (a harmonic trap with frequencies of 20 Hz and 650 Hz in the longitudinal and radial directions, respectively), and the atom-surface distance of 7.8 µm were kept constant.

The atomic distributions were recorded at each position, and the 2D map of 1D atomic line densities as a function of y-position was constructed (by integrating the column density images as described in the *BEC-M Measurement Procedure* section described above). We have recorded atomic probe scans under three current conditions: 1) without any current running through the CNT sample as a reference, 2) an identical scan with 50 mA flowing through the sample, 3) again with 100 mA through the sample. All data are compiled from three repeats of each to demonstrate reproducibility and increase the signal-to-noise ratio. The atomic density scans are shown in Figure 3 below. With higher current, the redistribution of atoms in the trap is even more pronounced.

The difference between the non-zero current and the reference scan for each of the two 50mA and 100mA cases were then used to calculate the additional magnetic field component generated by the sample. These final magnetic field scans are shown in Figure 5 of the main text.

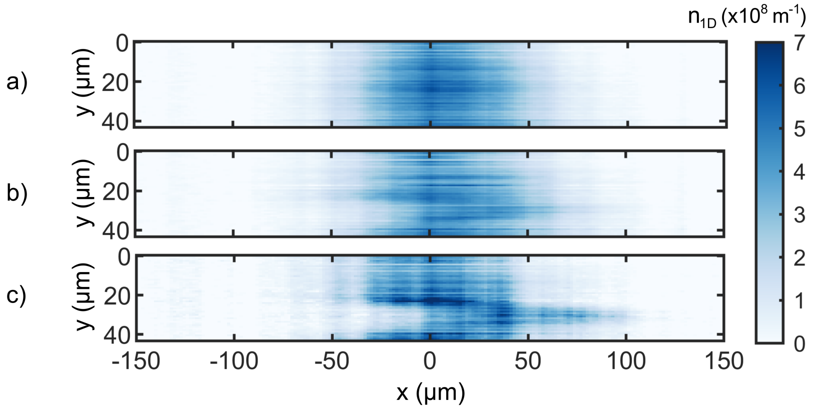

Figure 3: Measured atomic line density scans for varying currents through the carbon nanotube sample. (a) No current, for reference. (b) and (c) 50 mA and 100 mA through the sample, respectively.

References

[1] H. J. Metcalf and P. Van der Straten, *Laser cooling and trapping* (Springer Science & Business Media, 1999).

[2] J. Reichel and V. Vuletic, *Atom chips* (John Wiley & Sons, 2011).

[3] D. A. Smith et al., "Absorption imaging of ultracold atoms on atom chips", Opt. Express **19**, 8471–8485 (2011).

[4] F. Gerbier, "Quasi-1D Bose-Einstein condensates in the dimensional crossover regime", Europhys. Lett. **66**, 771 (2004).

[5] B. J. Roth, N. G. Sepulveda, and J. P. Wikswo Jr, "Using a magnetometer to image a two-dimensional current distribution", J. Appl. Phys. **65**, 361–372 (1989).

[6] A. T. Bellew et al., "Resistance of Single Ag Nanowire Junctions and Their Role in the Conductivity of Nanowire Networks", ACS Nano **9**, 11422–11429 (2015).
